# HPV Vaccine Delivery Strategies to Reach Out-of-School Girls in Low- and Middle-Income Countries: A Narrative Review

**DOI:** 10.3390/vaccines13050433

**Published:** 2025-04-22

**Authors:** Erica N. Rosser, Megan D. Wysong, Joseph G. Rosen, Rupali J. Limaye, Soim Park

**Affiliations:** 1 Department of International Health, Bloomberg School of Public Health, Johns Hopkins University, Baltimore, MD 21218, USA; mwysong1@jh.edu (M.D.W.); jrosen72@jhu.edu (J.G.R.); soim.park@jhu.edu (S.P.); 2 College of Public Health, George Mason University, Fairfax, VA 21985, USA

**Keywords:** HPV vaccination, out-of-school girls (OOS girls), low- and middle-income countries (LMICs), service delivery strategies

## Abstract

**Background/Objectives:** Low- and middle-income countries (LMICs) have the highest global burden of cervical cancer deaths. Human papillomavirus (HPV) vaccination is a key strategy for cervical cancer elimination, and in LMICs, global recommendations to vaccinate girls aged 9–14 years against HPV are generally implemented through school-based immunization platforms. Unfortunately, this strategy risks missing out-of-school (OOS) girls (i.e., girls not enrolled in formal schools). This narrative review maps the literature and synthesizes existing evidence on service delivery strategies for reaching OOS girls with HPV vaccination in LMICs. **Methods:** Using relevant databases, we conducted a narrative review of published, peer-reviewed literature to map and synthesize the existing evidence on effective service delivery strategies for reaching OOS girls with HPV vaccination in LMICs. **Results:** The 21 articles identified presented findings on strategies to reach OOS girls, with the most frequently cited strategies being facility-based and community outreach approaches. Authors also described community-based strategies used to identify and enumerate OOS girls, including peer tracing, church outreach initiatives, as well as partnerships with local groups (e.g., civil service organizations) and individuals (e.g., healthcare workers, teachers). The articles discussed barriers at the individual (e.g., lack of parental consent), facility/program delivery (e.g., lack of transportation for vaccines), and community (e.g., distance from homes to vaccination services) levels to HPV vaccine delivery, as well as solutions at the facility/program delivery (e.g., pilot programs) and community (e.g., multi-level partnerships) levels. **Conclusions:** Additional research is needed to evaluate implementation strategies targeting OOS girls with HPV vaccination. A better understanding of these strategies can provide valuable insights for HPV vaccine policymakers, healthcare providers, and program implementers.

## 1. Introduction

Human papillomavirus (HPV) is a common sexually transmitted infection that can progress to cervical cancer, a leading cause of death among women in low- and middle-income countries (LMICs). In 2022, LMICs accounted for 94% of global cervical cancer deaths [1]. HPV vaccination is a crucial preventive measure, and the World Health Organization (WHO) recommends vaccinating young girls against HPV before they become sexually active to reduce cervical cancer-related mortality and morbidity [1]. Globally, more than 70% of countries use school settings for HPV vaccine administration [2]. Schools provide an efficient way to reach girls who may have limited access to health services and school-based immunization platforms are widely employed in LMICs to vaccinate girls aged 9–14 years. However, this strategy risks leaving an important and substantial cohort of girls unvaccinated, that is, the approximately 119 million out-of-school (OOS) girls worldwide, defined as those not enrolled in formal schools [2,3,4,5,6,7].

Many factors contribute to girls’ OOS status, including poverty and gender inequities [4]. Research indicates that OOS girls are disproportionately located in rural areas with high rates of female school dropout [8]. These girls also often come from economically disadvantaged families, increasing their vulnerability to several health risks, including missed childhood vaccinations, early marriage, relationships with older partners, and sexually transmitted infections like HPV [9]. Furthermore, limited education for girls is linked to extreme poverty, high rates of teenage pregnancy and child mortality, and early sexual debut [8]. Recognizing missed HPV vaccinations in these girls threatens their future health, the WHO has emphasized the need to develop and implement strategies to reach and vaccinate OOS girls [9,10].

Identifying evidence-based strategies to reach OOS girls with HPV vaccination is an essential step to ensure equitable HPV vaccine coverage and reduce cervical cancer-related mortality and morbidity. However, there is limited peer-reviewed data reporting on the successes and challenges of delivering complementary HPV vaccination strategies to reach OOS girls in LMICs [11,12]. A better understanding of these strategies would provide valuable insights that can help policymakers, healthcare providers, and program implementers to increase HPV vaccine uptake. To address this gap in knowledge, the present narrative review aims to map and synthesize the existing evidence on effective service delivery strategies for reaching OOS girls with HPV vaccination in LMICs.

## 2. Materials and Methods

The specific aims of this review are to (1) identify the attributes and characteristics of service delivery strategies adopted and implemented to reach OOS girls for HPV vaccination in LMICs; (2) explore the potential effectiveness of these strategies for increasing HPV vaccination coverage among OOS girls, considering the contexts in which they are implemented; and (3) examine the implementation determinants (i.e., barriers and facilitators) of service delivery strategies for reaching OOS girls. Effectiveness was determined based on the study authors’ assessments, as reflected in their reporting on the success or failure of implemented strategies. This included, but was not limited to, changes in vaccination coverage, HPV vaccination awareness, the targeting of specific populations of OOS girls and cost effectiveness.

### 2.1. Search Strategy

A comprehensive search was conducted in four databases (i.e., PubMed, Embase, Global Index Medicus (GIM), and Scopus) of published, peer-reviewed literature using a combination of Medical Subject Headings (MeSH) terms and keywords related to HPV vaccination and service delivery strategies, OOS girls, and LMICs (see Appendix A). No date limits were used. The OOS girls and HPV vaccination hedges were developed through an iterative process which involved testing various search terms and analyzing the terminology used to describe OOS girls, HPV vaccination, and service delivery strategies within known relevant articles.

### 2.2. Study Selection

The Covidence online platform was used to facilitate the review process including screening and data extraction. After removing duplicates, a single reviewer (ENR) independently screened titles and abstracts retrieved from the search to identify potentially relevant articles. Full-text articles were retrieved for all studies deemed potentially relevant based on the initial screening and subsequently reviewed for eligibility using a predefined set of criteria. To ensure the accuracy of our study selection, a second reviewer (JGR) was consulted during the full-text review stage to resolve any questions regarding whether articles met the inclusion criteria. Uncertainties regarding article inclusion were resolved through a discussion between the two reviewers, with the second reviewer (JGR) independently reviewing the articles in question. The final decision on inclusion was made through consensus.

### 2.3. Collating, Summarizing, and Reporting

Data extraction was piloted on a subset of articles (*n* = 4) and then refined to ensure consistency. Data were extracted from the included studies using a standardized data extraction form that captured information on study characteristics, target population, service delivery strategies, reported effectiveness, as well as barriers and facilitators of strategy implementation. The extracted data were then charted, summarized, and narratively synthesized using the social ecological model to provide a comprehensive overview of the existing evidence base [13].

## 3. Results

### 3.1. Overview

**Figure 1:**
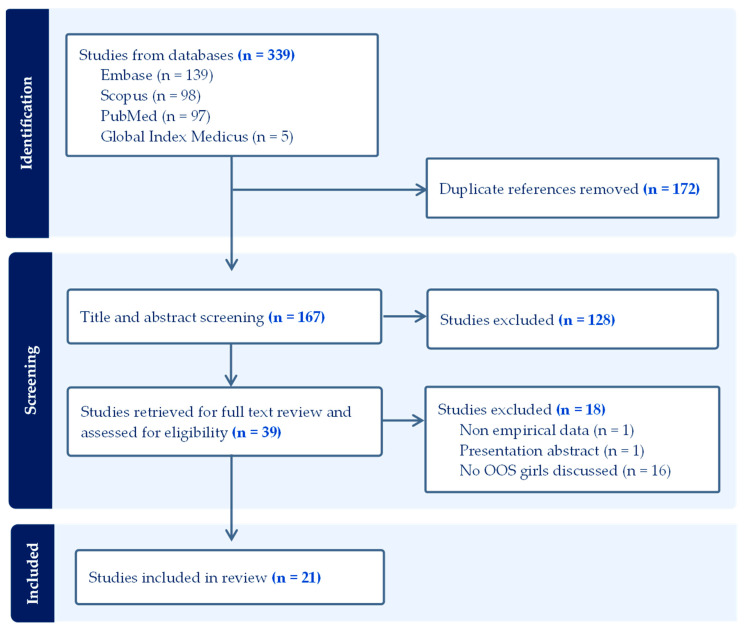
Out-of-school girls PRISMA flow diagram.

Figure 1 illustrates the results from database searching, title and abstract screening, and full-text review. A search of the four databases yielded 339 peer-reviewed articles. Next, 172 duplicates were removed, and 167 titles and abstracts were screened for relevance. In total, 39 full-text articles were identified and reviewed against inclusion and exclusion criteria. Reasons for exclusion included presentation of non-empirical data (*n* = 1), unavailable full-text (*n* = 1), and absent descriptions of OOS populations (*n* = 16). Finally, 21 publications were included in the literature corpus for data extraction and synthesis.

The 21 articles identified for this narrative review were published in 11 peer-reviewed journals between 2010 and 2024 (Table 1). Geographically, the studies represented a diverse range of LMICs, largely concentrated in African countries (*n* = 15). However, there was also representation from the WHO Americas (*n* = 2), European (*n* = 1), Southeast Asian (*n* = 3), and Western Pacific (*n* = 1) regions. Citations included a variety of study designs, the most common of which were qualitative (*n* = 8) and cross-sectional quantitative (*n* = 5) studies. Also present are descriptive program case studies or reports (*n* = 3), cost-effectiveness studies (*n* = 2), mixed-methods studies (*n* = 2), and cohort studies (*n* = 1). The HPV vaccination programs described in the studies were at various stages of implementation at the time the studies were conducted. Twelve studies reported on programs that were already in place, ranging from recent national introduction to more established/mature HPV vaccination programs. Four studies reported on subnational HPV vaccination pilots. For most of the studies conducted in multiple countries (*n* = 3), the status of national HPV vaccination programs varied across those countries. In one article, the status of the country’s HPV vaccination programs was unclear, and in another, it was not reported.

Table 1 provides a detailed breakdown of the included studies listed alphabetically by author. The table lists year of publication, study design and methodologies, implementation geographies, and the status of the national HPV vaccination program at the time of publication.

### 3.2. Key Findings by Study Aim

Key findings on service delivery strategies for reaching OOS girls with HPV vaccination in LMICs are presented in the following subsections: strategies to reach OOS girls, the effectiveness of these strategies in increasing HPV vaccine coverage, barriers to HPV vaccine delivery to OOS girls, and facilitators of HPV vaccine delivery to OOS girls.

#### 3.2.1. Strategies to Reach Out-of-School Girls with HPV Vaccine

Most of the articles (*n* = 14) specified on what scale HPV vaccination strategies (including those to reach OOS girls) were implemented. Seven articles listed HPV vaccination strategies as being implemented nationally [14,15,16,17,18,19,20] and six listed strategies as being implemented sub-nationally [21,22,23,24,25,26]. The study conducted by Gallagher et al. was implemented in numerous countries where the scale of implementation for vaccination programs varied widely [27].

The most frequently cited strategies implemented to reach OOS girls were facility-based [14,15,16,17,18,19,21,22,23,24,25,27,28] and outreach [14,16,17,18,19,21,22,25,26,27,28,29,30,31,32] approaches. Facility-based approaches leveraged existing healthcare infrastructure to offer HPV vaccines to eligible girls at local health facilities. For example, a formative research study in India recommended using Anganwadi centers (i.e., health facilities in Indian villages that provide basic services) to reach OOS girls [33]. Frequently, authors did not describe in detail what outreach activities consisted of, but a few referenced community outreach services or mobile sites along routes traveled by migratory groups which could be accessed by motor vehicles, motorcycles, bicycles, or camels [16,19,32]. Another study in Bhutan specified the benefit of using one-time vaccination catch-up campaigns that widened the age range of girls to be included for HPV vaccination, but also noted that this was primarily a school-based initiative [28]. Some of the authors also described strategies that were used to identify, enumerate, or reach OOS girls with HPV vaccination, including peer tracing [32], community door-to-door [30], community outreach integrated with child health days [29], church outreach programs [30], community posts [18], health facility listings of girls [16], approaching girls directly [16], public records [17], and unspecified community partnerships [20]. Articles also listed local civil society organizations [22], health ministry vaccinators [22], and teachers [21] as helping to identify girls who were absent or missed vaccination.

**Table 1:** Summary table of included articles.

First Author, Publication Year	Study Objectives	Study Design	Data Collection Methods	Countr(ies)	HPV Vaccine Program Status at the Time of Study/Intervention
Alonso et al., 2019 [21]	To estimate the costs associated with the demonstration programme of HPV vaccination during the 2014 cycle in the Manhica district and developed an alternative cost scenario for future implementation.	Mixed-method (Qualitative research and cost estimation)	Interviews; Secondary data (i.e., Documentation/record review)	Mozambique	Subnational pilot
Bangura et al., 2022 [20]	To describe cervical cancer disease burden and trends, HPV vaccination, screening, and health-related resources in Rwanda and Sierra Leone.	Case study	Secondary data (i.e., Documentation/record review)	Rwanda; Sierra Leone	Subnational pilot; National introduction
Casey et al., 2022 [18]	To review the introduction of the HPV vaccine into the routine immunization program in Senegal to better understand the successes, challenges, and lessons learned in program decision-making, planning, and implementation.	Qualitative research	Interviews; Secondary data (i.e., Documentation/record review); Observation of monitoring and supervision assessments	Senegal	National introduction
Dochez et al., 2017 [30]	To describe the activities and progress of a project to strengthen adolescent immunisation programmes in sub-Saharan Africa that was implemented from 2014–2016.	Case report	Primary documentation of activities	Botswana; Ethiopia; Kenya; Malawi; Mauritius; Mozambique; Namibia; Rwanda; Seychelles; South Africa; Swaziland; Tanzania; Uganda; Zambia; Zimbabwe;	Varied by country
Dorji et al., 2015 [28]	To characterize the implementation of an HPV vaccination program in Bhutan.	Case report	Secondary data (i.e., Documentation/record review)	Bhutan	National introduction
Doshi et al., 2022 [17]	To assess the awareness, feasibility, and acceptability of the HPV vaccination program in Senegal among key community stakeholders, including facility-based HCWs, community healthcare workers (cHCWs) delivering vaccines, school personnel, community leaders, and parents.	Cross-sectional study	Survey	Senegal	National introduction
Gallagher et al., 2017 [27]	To analyze coverage achieved in HPV vaccine demonstration projects and national programmes that had completed at least 6 months of implementation in LMICs in 2007–2016.	Qualitative research	Interviews; Secondary data (i.e., Documentation/record review)	Bolivia; Botswana; Brazil; Burkina Faso; Bhutan; Cambodia; Cameroon; Cote d’Ivoire; Ethiopia; Gambia; Georgia; Ghana; Guyana; Haiti; Honduras; India; Kenya; Kiribati; Lao PDR; Lesotho; Madagascar; Malawi; Mali; Moldova; Mongolia; Mozambique; Nepal; Niger; Papua New Guinea; Peru; Philippines; Rwanda; Senegal; Sierra Leone; Solomon Islands; South Africa; Tanzania; Thailand; Togo; Uganda; Uzbekistan; Vanuatu; Vietnam; Zambia; Zimbabwe	Varied by country
Hidle et al., 2018 [22]	To determine the cost of Zimbabwe’s HPV vaccination demonstration project.	Cost effectiveness	Secondary data (i.e., Documentation/record review)	Zimbabwe	Subnational pilot
Holroyd et al., 2022 [26]	To assess facilitators and barriers among OOS girls and proposed program characteristics to inform the design of pro-equity HPV vaccine delivery programs for OOS girls in Uttar Pradesh, India.	Qualitative research	FGD; Co-creation workshops	India	National introduction
Jacob et al., 2010 [33]	To investigate the sociocultural milieu, health system structures, and policy environments related to cervical cancer and HPV vaccines to generate information for the following three primary objectives relevant to government decision-making for potential future vaccine introduction:(i) design effective and appropriate HPV vaccine delivery systems for 10- to 14-year-old girls; (ii) design a communication strategy for HPV vaccine delivery; and (iii) devise an HPV vaccine advocacy strategy.	Qualitative research	Interviews; FGDs; Facility assessments; Vaccination session observations; Secondary data (i.e., Documentation/record review)	India	Subnational introduction
Kasonia et al., 2023 [29]	To determine whether the COVID-19 pandemic had an impact on essential primary healthcare services at public primary healthcare facilities.	Mixed-method	Interviews; Secondary data (i.e., Documentation/record review)	Democratic Republic of the Congo; Sierra Leone; Uganda	Varied by country
Li et al., 2022 [16]	To assess awareness, feasibility, and acceptability of the HPV vaccination program among health workers and community-level stakeholders.	Cross-sectional study	Survey	Tanzania	National introduction
Lubeya et al., 2024 [14]	To understand the implementation determinants of HPV vaccination among teachers and HCWs in Lusaka, Zambia using the consolidated framework for implementation research (CFIR) as a guiding framework.	Qualitative research	Interviews	Zambia	National introduction
Mphuru et al., 2022 [19]	To document the HPV vaccine introduction process to understand national scale-up and program implementation in Tanzania.	Qualitative research	Interviews; Secondary data (i.e., Documentation/record review)	Tanzania	National introduction
Msyamboza et al., 2017 [23]	To evaluate HPV vaccine coverage, lessons learned, and challenges identified during the first three years of implementation.	Cross-sectional study	Secondary data (i.e., Documentation/record review)	Malawi	Subnational pilot
Nabirye et al., 2020 [15]	To assess how the health systems is influencing uptake of HPV vaccine to inform policy for vaccine implementation and uptake in Mbale district, Eastern Uganda.	Cross-sectional study	Interviews; Survey	Uganda	National introduction
Riviere et al., 2021 [24]	To assess the success of a HPV vaccination program among adolescent girls aged 9–14 years in Haiti and to understand predictors of completion of a two-dose HPV vaccination series.	Cross-sectional study	Survey	Haiti	Subnational pilot
Rujumba et al., 2021 [34]	To explore barriers that prevent eligible girls from initiating or completing the recommended 2-dose HPV vaccine series in Oyam District, Northern Uganda.	Qualitative research	Interviews	Uganda	National introduction
Sayinzoga et al., 2020 [31]	To study determinants of vaccine coverage by birth cohort, province and vaccine dose.	Cohort study	Secondary data (i.e., Documentation/ record review)	Rwanda	National introduction
Simuyemba et al., 2024 [25]	To establish the cost to administer a single dose of the HPV vaccine as well as for full immunisation of two doses.	Cost effectiveness	Interviews; Secondary data (i.e., Documentation/record review)	Zambia	National introduction
Watson-Jones et al., 2015 [32]	To identify facilitators and barriers to HPV vaccination and potential acceptability of a future HPV vaccination programme amongst girls living in hard-to-reach populations in Kenya.	Qualitative research	Interviews; FGD; Situation assessment of community services	Kenya	National introduction

Abbreviations: HPV: human papillomavirus; LMICs: low- and middle-income countries; HCWs: healthcare workers; OOS: out-of-school; FGD: focus group discussion.

The stakeholders involved in implementing these strategies included facility-based health workers [14,15,17,18,19,21,28], community health workers [16,17,18,23,24,27], community-based organizations [19,30], civil service organizations [18,19,20], faith-based organizations [19,30], multi-lateral organizations [18,20,28], community leaders [16,17] or chiefs [23], community outreach workers (i.e., badjenu gox in Senegal) [17], and parents/guardians [16,17].

#### 3.2.2. Effectiveness of Strategies to Reach Out-of-School Girls with HPV Vaccine

Most articles provided few details about the characteristics of OOS girls reached, and there was minimal detail on the (perceived or measurable) effectiveness of strategies to vaccinate OOS girls, let alone any subpopulations of OOS girls. Two studies specifically referenced the benefit of integrating HPV vaccination services with existing health programs delivered at outreach clinics and posts that provide services for deworming, family planning, and HIV testing as a means to reach OOS girls, including pastoralists [15,32]. Several articles also discussed the importance of multimedia communication strategies to enhance HPV vaccination coverage and uptake [15,17,19,20,33]. These strategies included increasing the dissemination of print materials such as brochures, as well as conveying health messages through diverse channels such as radio, social media and television to enhance community awareness. Collaboration with key stakeholders including school personnel, healthcare workers (HCWs), and community leaders/groups was cited as a key strategy to bolster community trust. Reaching target audiences in key locations like health facilities, markets, water collection venues and churches were also deemed useful methods to enhance communication.

Writing about Senegal, Doshi et al. recommended that countries develop HPV-specific crisis management strategies to address potential misinformation about HPV vaccines [17]. Doshi et al. also recommended that given high rates of workforce turnover, continuous supportive supervision and refresher trainings would be helpful for implementation staff, specifically HCWs [17].

#### 3.2.3. Barriers to HPV Vaccine Delivery to Out-of-School Girls

Several of the articles (*n* = 9) noted barriers to implementing various HPV service delivery strategies, including those that could reach OOS girls [14,15,17,18,19,20,27,28,34]. Barriers cited at the individual, facility/program delivery, and community levels are presented in Table 2.

While most articles did not report on how these barriers were addressed, a few did report on how they managed vaccine hesitancy, misinformation, and rumors. For example, Dorji et al. reported that in response to media reports about a neighboring country (i.e., India) stopping HPV vaccination around the time of Bhutan’s national introduction, the Ministry of Health worked with the WHO to review safety data on the vaccine and generated press coverage reaffirming their intention to continue vaccination efforts [28]. In response to rumors circulating before and during HPV vaccine implementation, the Ministry of Health and Social Action in Senegal formed a committee to develop a plan focused on crisis communication, which included key messages in Wolof, the local language [17]. Casey et al. also reported on key adaptations to HPV vaccine implementation plans aimed at addressing vaccine hesitancy in Senegal. These adaptations included strengthening partnerships with community and religious leaders and expanding communication channels (e.g., social media, written press, radio, television) to target populations [18]. According to the authors, HPV vaccine hesitancy persisted in some areas and not all facilities initiated vaccination activities due to community resistance [18]. Mphuru et al. also reported on misinformation spread via the radio during national HPV vaccine introduction in Tanzania. In response, health communication specialists implemented a crisis communication plan, and persistent misinformation in limited areas was addressed through additional information and education activities, including re-sensitization of HCWs and community leaders [19]. While these solutions to rumors about HPV vaccination were generally not explicitly targeting OOS girls, they could nonetheless potentially benefit the OOS populations and other decision-makers in their periphery.

Casey et al. also reported that in response to a global HPV vaccine shortage, Senegal postponed their introduction and changed from a multi-age to single-age cohort, which induced confusion amongst HCWs and community members during delivery [18]. Teachers and HCWs involved in the HPV vaccination program in Zambia suggested that volunteers supporting vaccination campaigns receive incentives (i.e., money or attire for certain weather conditions) to ensure quality and continuity of vaccination efforts [14].

#### 3.2.4. Facilitators to HPV Vaccine Delivery to Out-of-School Girls

Several articles (*n* = 7) described facilitators of HPV vaccine service delivery strategies, including those that could reach OOS girls [14,15,18,19,20,27,32]. Facilitators cited at the facility/program delivery and community level are presented in Table 3. A few articles also noted that vaccine effectiveness, and global evidence on vaccine efficacy and safety facilitated national HPV vaccine introductions [14,18,32].

##### Program-Level Facilitators

In addition to citing well-known facilitators to vaccine delivery, including making vaccines free [14,20,32] and easy to access [14,32], articles highlighted other facilitators to HPV vaccine delivery. The opportunity to pilot HPV program delivery prior to scale-up was cited as a facilitator that made stakeholders more confident about their plans to introduce HPV vaccination in Zambia and Senegal, allowing them to anticipate and adapt to challenges (e.g., communication challenges) [14,18]. Microplanning was cited as a helpful activity for identifying not only resources needed for program delivery, but crucially for identifying eligible girls (both in-school and OOS girls) in Zambia and Tanzania [14,19]. Lubeya et al. cited HPV vaccination training sessions for implementors (e.g., teachers and HCWs) in Zambia as a key activity for informing those delivering and receiving the vaccine about its benefits [14]. Representatives from country HPV vaccine projects/programs in Gallagher et al. highlighted outreach activities (e.g., fixed/mobile sites in the community, door-to-door vaccine delivery) as being key for uptake among OOS girls specifically. The actors who participated in these activities (e.g., teachers, community health workers, HCWs) helped to identify OOS girls, follow-up with missed doses, and vaccinate girls [27].

Multiple studies conducted in sub-Saharan Africa cited service integration as an important HPV vaccine delivery strategy. In Rwanda, a strong vaccination and human resource system helped to achieve high coverage rates [20]. District health teams in Uganda asserted that integrating HPV vaccination with existing services allowed them to leverage current infrastructure to increase coverage [15]. Similarly, in Kenya, Ministry of Health staff perceived that integration with established outreach and mobile clinic services (using motor vehicles, motorcycles, bicycles, and camels) could bridge the gap with hard-to-reach populations, including OOS girls [32].

##### Community-Level Facilitators

Community sensitization via multiple channels was cited as a facilitator of vaccine delivery [14,27,32]. Articles noted that sensitization of all community members (including males who might influence girls’ uptake) not only helped to increase awareness of HPV vaccine programs (especially important to mobilize OOS girls), but also to dispel rumors and misconceptions circulating in communities.

Diverse partnerships were cited as playing an important role in HPV vaccine program success [14,18,20,32]. Articles noted that community and public–private collaborations helped with large-scale vaccine delivery. Their active participation throughout the implementation process helped to facilitate program success. These stakeholders included HCWs, teachers, religious and community leaders, civil service organizations, parents, adolescent girls, and media personnel. Government support for vaccine introduction was also cited as a facilitator [18,32].

## 4. Discussion

The current review identified a limited number of studies that specifically addressed strategies to reach OOS girls for HPV vaccination in LMICs. The most cited strategies included facility-based approaches and outreach activities. These approaches often involved implementation by facility- and community-based HCWs, as well as community leaders, and other stakeholders including community-based and civil service organizations. However, the details of these outreach activities were often insufficiently described, and in general, authors provided limited evidence on the effectiveness of these strategies (e.g., improvements in HPV vaccination coverage) specifically for reaching OOS girls. Future research is needed to characterize the implementation and effectiveness of HPV vaccine delivery strategies tailored to OOS girls in LMICs.

This review also identified several facilitators and barriers to implementing service delivery strategies enhancing HPV vaccination uptake among girls, including OOS girls. Challenges to implementing these strategies include a lack of awareness about HPV vaccination among both parents and HCWs, vaccine hesitancy due to misinformation, as well as logistical hurdles like vaccine supply shortages and insufficient resources. Facilitators included community sensitization and outreach activities, diverse partnerships, and service integration with existing health programs. Barriers and facilitators often materialized at multiple levels and were synergistic; for example, inadequate training of HCWs (facility-level barrier) can lead to poor communication with parents about the vaccine (individual-level barrier), ultimately reducing vaccine uptake among OOS girls. Similarly, a lack of parental knowledge about HPV vaccination (individual-level barrier) can be addressed through community sensitization activities (community-level facilitator), but the effectiveness of these activities can be hampered by limited resources (program-level barrier) or gendered norms/expectations constraining HPV vaccination uptake among OOS girls (community-level barriers). These factors highlight the need for comprehensive approaches that address barriers at multiple levels.

Articles included in this review inconsistently defined “out-of-school” status when describing OOS girls, revealing underlying heterogeneities in this population but leading to inconsistent application of the term across the literature. Other researchers have also highlighted this phenomenon. Tsu et al., noted that due to the poorly defined parameters of OOS girls, the term is used to describe both girls absent from school at the time of vaccination and girls who are not enrolled in school [11]. While Ozawa et al. focus on distinguishing between “hard-to-reach” (a group into which many OOS girls commonly fall) and “hard-to-vaccinate” population (i.e., populations that have access to vaccination but delay or refuse), their work emphasizes the importance of having a consistent definition to facilitate sharing knowledge and harmonizing tailored intervention development. For example, they assert that ‘‘hard-to-reach” emphasizes vaccine delivery challenges, while ‘‘hard-to-vaccinate” focuses on vaccine uptake and acceptance, a distinction which can help to determine appropriate interventions for each group [35]. A clearer definition of OOS girls is urgently needed. Girls absent during school-based vaccination campaigns differ from girls who are completely out-of-school and face specific socio-structural barriers such as residing in an area marred by conflict or belonging to a nomadic population. Clarifying parameters of “OOS girls” in the context of HPV vaccination programming is essential to addressing their discrete needs.

### Limitations

This narrative review has several limitations. Firstly, we did not identify any implementation strategies specifically or exclusively targeting OOS girls. This review also focused on English-language literature and may have missed relevant research published in other languages. The cross-sectional nature of many studies included in the review limits our ability to draw conclusions about temporal relationships between implementation strategy delivery and HPV vaccination outcomes. Finally, the concept of OOS girls was not always explicitly mentioned in articles. Despite efforts to conduct a comprehensive search of available literature, the search strategy may have inadvertently excluded relevant studies. As stated previously, we employed several keywords identified in known relevant articles to mitigate this challenge.

## 5. Conclusions

This is among the first review studies mapping the literature on strategies to reach OOS girls with HPV vaccination in LMICs. Further research is also needed to explore how to best address the barriers and leverage the facilitators identified in this review to improve HPV vaccination coverage among OOS girls in LMICs. Additional research should include studies that evaluate how successfully different implementation strategies specifically designed to reach OOS girls contribute to increased levels of HPV vaccination coverage among this population. Researchers should also investigate the success of implementation outcomes for HPV vaccination strategies targeting OOS girls such as rates of vaccine acceptance. Evidence on the cost and feasibility of implementing different strategies as well as the long-term sustainability of interventions in different settings would be a valuable resource for decision-makers and implementers needing to select effective strategies for increasing HPV vaccination coverage among OOS girls.

## Figures and Tables

**Table 2:** Barriers to HPV vaccine service delivery.

Level	Barriers
**Individual**	Lack of parental consent [14] Inadequate knowledge about or sensitization to HPV vaccines (e.g., suboptimal parental awareness, limited knowledge of vaccination activities) [14,18,32,34] Mobility, migration, or change of residence or school district [34] Absenteeism or school attrition [34] Fear of injection pain or side effects [32,34] Discouragement from vaccination by caregivers or peers [34] Busy schedules and gendered nature of household duties [32,34]
**Facility/program**	Inadequate training on HPV vaccine and national HPV vaccination policy (e.g., unawareness of vaccination strategies tailored to OOS girls) [15,34] Lack of coherent plans to reach OOS girls with HPV vaccine [15,34] Vaccine supply shortages and inadequacies of cold chain infrastructure [15,34] Insufficient transportation resources for vaccination outreach [14,34] Lack of staff incentives [14,34] Insufficient facility staff [20,34] Protests and strikes by the healthcare workforce [18] Challenges in identifying OOS girls [27,30]
**Community**	Rumors and misconceptions about HPV vaccine and vaccination (e.g., infertility [most common], vaccine increases cancer risk, safety concerns, vaccine promotes early sexual activity, vaccine is experimental) [14,17,18,19,28,32,34] Limited social mobilization and community engagement activities [14,34] Socio-cultural gender norms, including religious beliefs [34] Distances from homes to vaccination services [32]

**Table 3:** Facilitators to HPV vaccine service delivery.

Level	Facilitators
**Facility/program**	Pilot programs [14,18] Microplanning activities [14,19] Training activities [14] Outreach activities [27] Service integration [15,20,32] Free vaccines [14,20,32] Accessible vaccines [14,32]
**Community**	Community sensitization using various platforms [14,27,32] Government endorsement [18,32] Multi-level partnerships [14,18,20,32]

## Appendix A

**Table A1:** Database search results.

Concepts	Number of Articles Retrieved *(Search Date: 13 March 2024)*				
	PubMed	Embase	Global Index Medicus (GIM)	Scopus	Total
**1—HPV**	18,023	28,650 *	949	--	
**2—OOS**	31,500	41,349 **	11,834	--	
**3—LMIC**	2,635,390	3,189,675	N/A ***	--	
**1 & 2 & 3**	97	139	5	98 ****	**339**

***** Embase searches 1, 2, and 3. ****** Embase searches 4 and 5. ******* GIM provides access to literature produced by and within LMICs. ******** All search terms entered into Advanced Search on Scopus.

Search Strategy Implemented for Each Database

**PUBMED Search terms:**

**HPV vaccination/immunization**

“Papillomavirus Vaccines”[Mesh] OR “hpv vaccination”[tiab:~3] OR “hpv immunisation”[tiab:~3] OR “hpv immunization”[tiab:~3] OR “hpv vaccinations”[tiab:~3] OR “hpv immunisations”[tiab:~3] OR “hpv immunizations”[tiab:~3] OR ((HPV[tiab] OR “papillomavirus vaccine*”[tiab] OR “papilloma virus vaccine*”[tiab] OR Gardasil*[tw] OR Cervarix[tw] OR Cecolin[tw] OR Walrinvax[tw] OR Cervava*[tw]) AND (“vaccine delivery”[tiab] OR “administration”[tiab] OR “strateg*”[tiab] OR implementation[tiab])) OR ((HPV[tiab] OR “human papillomavirus*”[tiab] OR “human papilloma virus*”[tiab]) AND (“immunization programs”[mesh] OR “immunisation program*”[tiab] OR “immunization program*”[tiab]))

***
  OOS girls
  ***
“not school based”[tiab:~3] OR “non-school based”[tiab] OR “nonschool based”[tiab] OR unschooled[tiab] OR “out of school”[tiab:~3] OR “out of schools”[tiab:~3] OR “outreach”[tiab] OR “out reach”[tiab] OR “facility based”[tiab:~3] OR “unenrolled”[tiab] OR “un-enrolled”[tiab] OR “hard to reach”[tiab]

***
  LMICs
  ***
(((afghanistan[MeSH] OR albania[MeSH] OR algeria[MeSH] OR american samoa[MeSH] OR angola[MeSH] OR antigua and barbuda[MeSH] OR argentina[MeSH] OR armenia[MeSH] OR aruba[MeSH] OR azerbaijan[MeSH] OR bahrain[MeSH] OR bangladesh[MeSH] OR barbados[MeSH] OR republic of belarus[MeSH] OR belize[MeSH] OR benin[MeSH] OR bhutan[MeSH] OR bolivia[MeSH] OR bosnia and herzegovina[MeSH] OR botswana[MeSH] OR brazil[MeSH] OR bulgaria[MeSH] OR burkina faso[MeSH] OR burundi[MeSH] OR cabo verde[MeSH] OR cambodia[MeSH] OR cameroon[MeSH] OR central african republic[MeSH] OR chad[MeSH] OR chile[MeSH] OR china[MeSH] OR colombia[MeSH] OR comoros[MeSH] OR democratic republic of the congo[MeSH] OR congo[MeSH] OR costa rica[MeSH] OR cote d’ivoire[MeSH] OR croatia[MeSH] OR cuba[MeSH] OR cyprus[MeSH] OR czech republic[MeSH] OR djibouti[MeSH] OR dominica[MeSH] OR dominican republic[MeSH] OR ecuador[MeSH] OR egypt[MeSH] OR el salvador[MeSH] OR equatorial guinea[MeSH] OR eritrea[MeSH] OR estonia[MeSH] OR eswatini[Mesh] OR swaziland[MeSH] OR ethiopia[MeSH] OR fiji[MeSH] OR gabon[MeSH] OR gambia[MeSH] OR “georgia (republic)”[MeSH] OR ghana[MeSH] OR gibraltar[MeSH] OR grenada[MeSH] OR guam[MeSH] OR guatemala[MeSH] OR guinea[MeSH] OR guinea bissau[MeSH] OR guyana[MeSH] OR haiti[MeSH] OR honduras[MeSH] OR india[MeSH] OR indonesia[MeSH] OR iran[MeSH] OR iraq[MeSH] OR jamaica[MeSH] OR jordan[MeSH] OR kazakhstan[MeSH] OR kenya[MeSH] OR “democratic people’s republic of korea”[MeSH] OR republic of korea[MeSH] OR kosovo[MeSH] OR kyrgyzstan[MeSH] OR laos[MeSH] OR latvia[MeSH] OR lebanon[MeSH] OR lesotho[MeSH] OR liberia[MeSH] OR libya[MeSH] OR lithuania[MeSH] OR macau[MeSH] OR “republic of north macedonia”[MeSH] OR madagascar[MeSH] OR malawi[MeSH] OR malaysia[MeSH] OR indian ocean islands[MeSH] OR mali[MeSH] OR malta[MeSH] OR micronesia[MeSH] OR palau[MeSH] OR mauritania[MeSH] OR mauritius[MeSH] OR mexico[MeSH] OR moldova[MeSH] OR mongolia[MeSH] OR montenegro[MeSH] OR morocco[MeSH] OR mozambique[MeSH] OR myanmar[MeSH] OR namibia[MeSH] OR nepal[MeSH] OR netherlands antilles[MeSH] OR nicaragua[MeSH] OR niger[MeSH] OR nigeria[MeSH] OR oman[MeSH] OR pakistan[MeSH] OR panama[MeSH] OR papua new guinea[MeSH] OR paraguay[MeSH] OR peru[MeSH] OR philippines[MeSH] OR poland[MeSH] OR portugal[MeSH] OR puerto rico[MeSH] OR romania[MeSH] OR russia[MeSH] OR rwanda[MeSH] OR samoa[MeSH] OR sao tome and principe[MeSH] OR saudi arabia[MeSH] OR senegal[MeSH] OR serbia[MeSH] OR seychelles[MeSH] OR sierra leone[MeSH] OR slovakia[MeSH] OR slovenia[MeSH] OR melanesia[MeSH] OR somalia[MeSH] OR south africa[MeSH] OR south sudan[MeSH] OR sri lanka[MeSH] OR saint kitts and nevis[MeSH] OR saint lucia[MeSH] OR saint vincent and the grenadines[MeSH] OR sudan[MeSH] OR suriname[MeSH] OR syria[MeSH] OR tajikistan[MeSH] OR tanzania[MeSH] OR thailand[MeSH] OR timor leste[MeSH] OR togo[MeSH] OR tonga[MeSH] OR trinidad and tobago[MeSH] OR tunisia[MeSH] OR “turkey”[MeSH] OR turkmenistan[MeSH] OR uganda[MeSH] OR ukraine[MeSH] OR uruguay[MeSH] OR uzbekistan[MeSH] OR vanuatu[MeSH] OR venezuela[MeSH] OR vietnam[MeSH] OR middle east[MeSH] OR yemen[MeSH] OR yugoslavia[MeSH] OR zambia[MeSH] OR zimbabwe[MeSH] OR africa south of the sahara[MeSH] OR africa, central[MeSH] OR africa, northern[MeSH] OR africa, southern[MeSH] OR africa, eastern[MeSH] OR africa, western[MeSH] OR west indies[MeSH] OR indian ocean islands[MeSH] OR caribbean region[MeSH] OR central america[MeSH] OR latin america[MeSH] OR south america[MeSH] OR asia, central[MeSH] OR asia, northern[MeSH] OR asia, southeastern[MeSH] OR asia, western[MeSH] OR europe, eastern[MeSH] OR developing countries[MeSH]) OR (afghanistan[Text Word] OR albania[Text Word] OR algeria[Text Word] OR american samoa[Text Word] OR angola[Text Word] OR antigua[Text Word] OR barbuda[Text Word] OR argentina[Text Word] OR armenia[Text Word] OR armenian[Text Word] OR aruba[Text Word] OR azerbaijan[Text Word] OR bahrain[Text Word] OR bangladesh[Text Word] OR barbados[Text Word] OR belarus[Text Word] OR byelarus[Text Word] OR belorussia[Text Word] OR byelorussian[Text Word] OR belize[Text Word] OR british honduras[Text Word] OR benin[Text Word] OR dahomey[Text Word] OR bhutan[Text Word] OR bolivia[Text Word] OR bosnia[Text Word] OR herzegovina[Text Word] OR botswana[Text Word] OR bechuanaland[Text Word] OR brazil[Text Word] OR brasil[Text Word] OR bulgaria[Text Word] OR burkina faso[Text Word] OR burkina fasso[Text Word] OR upper volta[Text Word] OR burundi[Text Word] OR urundi[Text Word] OR cabo verde[Text Word] OR cape verde[Text Word] OR cambodia[Text Word] OR kampuchea[Text Word] OR khmer republic[Text Word] OR cameroon[Text Word] OR cameron[Text Word] OR cameroun[Text Word] OR central african republic[Text Word] OR ubangi shari[Text Word] OR chad[Text Word] OR chile[Text Word] OR china[Text Word] OR colombia[Text Word] OR comoros[Text Word] OR comoro islands[Text Word] OR mayotte[Text Word] OR congo[Text Word] OR zaire[Text Word] OR costa rica[Text Word] OR cote d’ivoire[Text Word] OR cote d’ivoire[Text Word] OR cote d’ivoire[Text Word] OR ivory coast[Text Word] OR croatia[Text Word] OR cuba[Text Word] OR cyprus[Text Word] OR czech republic[Text Word] OR czechoslovakia[Text Word] OR djibouti[Text Word] OR french somaliland[Text Word] OR dominica[Text Word] OR dominican republic[Text Word] OR ecuador[Text Word] OR egypt[Text Word] OR united arab republic[Text Word] OR el salvador[Text Word] OR equatorial guinea[Text Word] OR spanish guinea[Text Word] OR eritrea[Text Word] OR estonia[Text Word] OR eswatini[Text Word] OR swaziland[Text Word] OR ethiopia[Text Word] OR fiji[Text Word] OR gabon[Text Word] OR gabonese republic[Text Word] OR gambia[Text Word] OR georgia[Text Word] OR georgian[Text Word] OR ghana[Text Word] OR gold coast[Text Word] OR gibraltar[Text Word] OR grenada[Text Word] OR guam[Text Word] OR guatemala[Text Word] OR guinea[Text Word] OR guyana[Text Word] OR guiana[Text Word] OR haiti[Text Word] OR hispaniola[Text Word] OR honduras[Text Word] OR india[Text Word] OR indonesia[Text Word] OR timor[Text Word] OR iran[Text Word] OR iraq[Text Word] OR isle of man[Text Word] OR jamaica[Text Word] OR jordan[Text Word] OR kazakhstan[Text Word] OR kazakh[Text Word] OR kenya[Text Word] OR korea[Text Word] OR kosovo[Text Word] OR kyrgyzstan[Text Word] OR kirghizia[Text Word] OR kirgizstan[Text Word] OR kyrgyz republic[Text Word] OR kirghiz[Text Word] OR laos[Text Word] OR lao pdr[Text Word] OR lao people’s democratic republic[Text Word] OR latvia[Text Word] OR lebanon[Text Word] OR lesotho[Text Word] OR basutoland[Text Word] OR liberia[Text Word] OR libya[Text Word] OR libyan arab jamahiriya[Text Word] OR lithuania[Text Word] OR macau[Text Word] OR macao[Text Word] OR macedonia[Text Word] OR madagascar[Text Word] OR malagasy republic[Text Word] OR malawi[Text Word] OR nyasaland[Text Word] OR malaysia[Text Word] OR maldives[Text Word] OR indian ocean[Text Word] OR mali[Text Word] OR malta[Text Word] OR micronesia[Text Word] OR kiribati[Text Word] OR marshall islands[Text Word] OR nauru[Text Word] OR northern mariana islands[Text Word] OR palau[Text Word] OR tuvalu[Text Word] OR mauritania[Text Word] OR mauritius[Text Word] OR mexico[Text Word] OR moldova[Text Word] OR moldovian[Text Word] OR mongolia[Text Word] OR montenegro[Text Word] OR morocco[Text Word] OR ifni[Text Word] OR mozambique[Text Word] OR portuguese east africa[Text Word] OR myanmar[Text Word] OR burma[Text Word] OR namibia[Text Word] OR nepal[Text Word] OR netherlands antilles[Text Word] OR nicaragua[Text Word] OR niger[Text Word] OR nigeria[Text Word] OR oman[Text Word] OR muscat[Text Word] OR pakistan[Text Word] OR panama[Text Word] OR papua new guinea[Text Word] OR paraguay[Text Word] OR peru[Text Word] OR philippines[Text Word] OR philipines[Text Word] OR phillipines[Text Word] OR phillippines[Text Word] OR poland[Text Word] OR polish people’s republic[Text Word] OR portugal[Text Word] OR portuguese republic[Text Word] OR puerto rico[Text Word] OR romania[Text Word] OR russia[Text Word] OR russian federation[Text Word] OR ussr[Text Word] OR soviet union[Text Word] OR union of soviet socialist republics[Text Word] OR rwanda[Text Word] OR ruanda[Text Word] OR samoa[Text Word] OR pacific islands[Text Word] OR polynesia[Text Word] OR samoan islands[Text Word] OR sao tome and principe[Text Word] OR saudi arabia[Text Word] OR senegal[Text Word] OR serbia[Text Word] OR seychelles[Text Word] OR sierra leone[Text Word] OR slovakia[Text Word] OR slovak republic[Text Word] OR slovenia[Text Word] OR melanesia[Text Word] OR solomon island[Text Word] OR solomon islands[Text Word] OR norfolk island[Text Word] OR somalia[Text Word] OR south africa[Text Word] OR south sudan[Text Word] OR sri lanka[Text Word] OR ceylon[Text Word] OR saint kitts and nevis[Text Word] OR st kitts and nevis[Text Word] OR saint lucia[Text Word] OR st lucia[Text Word] OR saint vincent[Text Word] OR st vincent[Text Word] OR grenadines[Text Word] OR sudan[Text Word] OR suriname[Text Word] OR surinam[Text Word] OR syria[Text Word] OR syrian arab republic[Text Word] OR tajikistan[Text Word] OR tadjikistan[Text Word] OR tadzhikistan[Text Word] OR tadzhik[Text Word] OR tanzania[Text Word] OR tanganyika[Text Word] OR thailand[Text Word] OR siam[Text Word] OR timor leste[Text Word] OR east timor[Text Word] OR togo[Text Word] OR togolese republic[Text Word] OR tonga[Text Word] OR trinidad[Text Word] OR tobago[Text Word] OR tunisia[Text Word] OR turkey[Text Word] OR turkmenistan[Text Word] OR turkmen[Text Word] OR uganda[Text Word] OR ukraine[Text Word] OR uruguay[Text Word] OR uzbekistan[Text Word] OR uzbek[Text Word] OR vanuatu[Text Word] OR new hebrides[Text Word] OR venezuela[Text Word] OR vietnam[Text Word] OR viet nam[Text Word] OR middle east[Text Word] OR west bank[Text Word] OR gaza[Text Word] OR palestine[Text Word] OR yemen[Text Word] OR yugoslavia[Text Word] OR zambia[Text Word] OR zimbabwe[Text Word] OR northern rhodesia[Text Word] OR global south[Text Word] OR africa south of the sahara[Text Word] OR sub saharan africa[Text Word] OR subsaharan africa[Text Word] OR central africa[Text Word] OR north africa[Text Word] OR northern africa[Text Word] OR magreb[Text Word] OR maghrib[Text Word] OR sahara[Text Word] OR southern africa[Text Word] OR east africa[Text Word] OR eastern africa[Text Word] OR west africa[Text Word] OR western africa[Text Word] OR west indies[Text Word] OR indian ocean islands[Text Word] OR caribbean[Text Word] OR central america[Text Word] OR latin america[Text Word] OR south america[Text Word] OR central asia[Text Word] OR north asia[Text Word] OR northern asia[Text Word] OR southeastern asia[Text Word] OR south eastern asia[Text Word] OR southeast asia[Text Word] OR south east asia[Text Word] OR western asia[Text Word] OR east europe[Text Word] OR eastern europe[Text Word] OR developing country[Text Word] OR developing countries[Text Word] OR developing nation[Text Word] OR developing nations[Text Word] OR developing population[Text Word] OR developing populations[Text Word] OR developing world[Text Word] OR less developed country[Text Word] OR less developed countries[Text Word] OR less developed nation[Text Word] OR less developed nations[Text Word] OR less developed world[Text Word] OR lesser developed countries[Text Word] OR lesser developed nations[Text Word] OR under developed country[Text Word] OR under developed countries[Text Word] OR under developed nations[Text Word] OR under developed world[Text Word] OR underdeveloped country[Text Word] OR underdeveloped countries[Text Word] OR underdeveloped nation[Text Word] OR underdeveloped nations[Text Word] OR underdeveloped population[Text Word] OR underdeveloped populations[Text Word] OR underdeveloped world[Text Word] OR middle income country[Text Word] OR middle income countries[Text Word] OR middle income nation[Text Word] OR middle income nations[Text Word] OR middle income population[Text Word] OR middle income populations[Text Word] OR low income country[Text Word] OR low income countries[Text Word] OR low income nation[Text Word] OR low income nations[Text Word] OR low income population[Text Word] OR low income populations[Text Word] OR lower income country[Text Word] OR lower income countries[Text Word] OR lower income nations[Text Word] OR lower income population[Text Word] OR lower income populations[Text Word] OR underserved countries[Text Word] OR underserved nations[Text Word] OR underserved population[Text Word] OR underserved populations[Text Word] OR under served population[Text Word] OR under served populations[Text Word] OR deprived countries[Text Word] OR deprived population[Text Word] OR deprived populations[Text Word] OR poor country[Text Word] OR poor countries[Text Word] OR poor nation[Text Word] OR poor nations[Text Word] OR poor population[Text Word] OR poor populations[Text Word] OR poor world[Text Word] OR poorer countries[Text Word] OR poorer nations[Text Word] OR poorer population[Text Word] OR poorer populations[Text Word] OR developing economy[Text Word] OR developing economies[Text Word] OR less developed economy[Text Word] OR less developed economies[Text Word] OR underdeveloped economies[Text Word] OR middle income economy[Text Word] OR middle income economies[Text Word] OR low income economy[Text Word] OR low income economies[Text Word] OR lower income economies[Text Word] OR low gdp[Text Word] OR low gnp[Text Word] OR low gross domestic[Text Word] OR low gross national[Text Word] OR lower gdp[Text Word] OR lower gross domestic[Text Word] OR lmic[Text Word] OR lmics[Text Word] OR third world[Text Word] OR lami country[Text Word] OR lami countries[Text Word] OR transitional country[Text Word] OR transitional countries[Text Word] OR emerging economies[Text Word] OR emerging nation[Text Word] OR emerging nations[Text Word])))

**Embase Search terms:**
***HPV vaccination/immunization***
***#1***. (‘preventive health service’/exp OR ‘immuni?ation program*’) AND (hpv:ti,ab,kw OR ‘human papillomavirus*’:ti,ab,kw OR ‘human papilloma virus*’:ti,ab,kw)
OR
**#2.** (hpv:ti,ab,kw OR ‘papillomavirus vaccine*’:ti,ab,kw OR ‘papilloma virus vaccine*’:ti,ab,kw OR gardasil*:ti,ab,kw OR cervarix:ti,ab,kw OR cecolin:ti,ab,kw OR walrinvax:ti,ab,kw OR cervava*:ti,ab,kw) AND (‘vaccine delivery’:ti,ab,kw OR ‘administration’:ti,ab,kw OR ‘strateg*’:ti,ab,kw OR implementation:ti,ab,kw)
OR
**#3.** (hpv NEAR/3 vaccinat*) OR (hpv NEAR/3 immuni*) OR ‘human papilloma virus vaccine’/exp

***OOS girls***
**#4.** ‘out of school youths’/exp OR ‘outreach’/exp OR ‘outreach program’/exp
OR
**#5.** (‘not’ NEAR/3 school NEAR/3 based) OR ‘non-school based’:ti,ab,kw OR ‘nonschool based’:ti,ab,kw OR unschooled:ti,ab,kw OR (‘out of’ NEAR/3 school*) OR ‘outreach’:ti,ab,kw OR ‘out reach’:ti,ab,kw OR (facility NEAR/3 based) OR ‘unenrolled’:ti,ab,kw OR ‘un-enrolled’:ti,ab,kw OR ‘hard to reach’:ti,ab,kw

***LMICs***
**#6.** ‘developing country’:ab,ti OR ‘developing countries’:ab,ti OR ‘developing nation’:ab,ti OR ‘developing nations’:ab,ti OR ‘developing population’:ab,ti OR ‘developing populations’:ab,ti OR ‘developing world’:ab,ti OR ‘less developed country’:ab,ti OR ‘less developed countries’:ab,ti OR ‘less developed nation’:ab,ti OR ‘less developed nations’:ab,ti OR ‘less developed population’:ab,ti OR ‘less developed populations’:ab,ti OR ‘less developed world’:ab,ti OR ‘lesser developed country’:ab,ti OR ‘lesser developed countries’:ab,ti OR ‘lesser developed nation’:ab,ti OR ‘lesser developed nations’:ab,ti OR ‘lesser developed population’:ab,ti OR ‘lesser developed populations’:ab,ti OR ‘lesser developed world’:ab,ti OR ‘under developed country’:ab,ti OR ‘under developed countries’:ab,ti OR ‘under developed nation’:ab,ti OR ‘under developed nations’:ab,ti OR ‘under developed population’:ab,ti OR ‘under developed populations’:ab,ti OR ‘under developed world’:ab,ti OR ‘underdeveloped country’:ab,ti OR ‘underdeveloped countries’:ab,ti OR ‘underdeveloped nation’:ab,ti OR ‘underdeveloped nations’:ab,ti OR ‘underdeveloped population’:ab,ti OR ‘underdeveloped populations’:ab,ti OR ‘underdeveloped world’:ab,ti OR ‘middle income country’:ab,ti OR ‘middle income countries’:ab,ti OR ‘middle income nation’:ab,ti OR ‘middle income nations’:ab,ti OR ‘middle income population’:ab,ti OR ‘middle income populations’:ab,ti OR ‘low income country’:ab,ti OR ‘low income countries’:ab,ti OR ‘low income nation’:ab,ti OR ‘low income nations’:ab,ti OR ‘low income population’:ab,ti OR ‘low income populations’:ab,ti OR ‘lower income country’:ab,ti OR ‘lower income countries’:ab,ti OR ‘lower income nation’:ab,ti OR ‘lower income nations’:ab,ti OR ‘lower income population’:ab,ti OR ‘lower income populations’:ab,ti OR ‘underserved country’:ab,ti OR ‘underserved countries’:ab,ti OR ‘underserved nation’:ab,ti OR ‘underserved nations’:ab,ti OR ‘underserved population’:ab,ti OR ‘underserved populations’:ab,ti OR ‘underserved world’:ab,ti OR ‘under served country’:ab,ti OR ‘under served countries’:ab,ti OR ‘under served nation’:ab,ti OR ‘under served nations’:ab,ti OR ‘under served population’:ab,ti OR ‘under served populations’:ab,ti OR ‘under served world’:ab,ti OR ‘deprived country’:ab,ti OR ‘deprived countries’:ab,ti OR ‘deprived nation’:ab,ti OR ‘deprived nations’:ab,ti OR ‘deprived population’:ab,ti OR ‘deprived populations’:ab,ti OR ‘deprived world’:ab,ti OR ‘poor country’:ab,ti OR ‘poor countries’:ab,ti OR ‘poor nation’:ab,ti OR ‘poor nations’:ab,ti OR ‘poor population’:ab,ti OR ‘poor populations’:ab,ti OR ‘poor world’:ab,ti OR ‘poorer country’:ab,ti OR ‘poorer countries’:ab,ti OR ‘poorer nation’:ab,ti OR ‘poorer nations’:ab,ti OR ‘poorer population’:ab,ti OR ‘poorer populations’:ab,ti OR ‘poorer world’:ab,ti OR ‘developing economy’:ab,ti OR ‘developing economies’:ab,ti OR ‘less developed economy’:ab,ti OR ‘less developed economies’:ab,ti OR ‘lesser developed economy’:ab,ti OR ‘lesser developed economies’:ab,ti OR ‘under developed economy’:ab,ti OR ‘under developed economies’:ab,ti OR ‘underdeveloped economy’:ab,ti OR ‘underdeveloped economies’:ab,ti OR ‘middle income economy’:ab,ti OR ‘middle income economies’:ab,ti OR ‘low income economy’:ab,ti OR ‘low income economies’:ab,ti OR ‘lower income economy’:ab,ti OR ‘lower income economies’:ab,ti OR ‘low gdp’:ab,ti OR ‘low gnp’:ab,ti OR ‘low gross domestic’:ab,ti OR ‘low gross national’:ab,ti OR ‘lower gdp’:ab,ti OR ‘lower gnp’:ab,ti OR ‘lower gross domestic’:ab,ti OR ‘lower gross national’:ab,ti OR lmic:ab,ti OR lmics:ab,ti OR ‘third world’:ab,ti OR ‘lami country’:ab,ti OR ‘lami countries’:ab,ti OR ‘transitional country’:ab,ti OR ‘transitional countries’:ab,ti OR africa:ti,ab OR asia:ti,ab OR caribbean:ti,ab OR ‘west indies’:ti,ab OR ‘south america’:ti,ab OR ‘latin america’:ti,ab OR ‘central america’:ti,ab OR ‘atlantic islands’:ab,ti OR ‘commonwealth of independent states’:ab,ti OR ‘pacific islands’:ab,ti OR ‘indian ocean islands’:ab,ti OR ‘eastern europe’:ab,ti OR afghanistan:ti,ab OR albania:ti,ab OR algeria:ti,ab OR ‘american samoa’:ti,ab OR angola:ti,ab OR antigua:ti,ab OR barbuda:ti,ab OR argentina:ti,ab OR armenia:ti,ab OR armenian:ti,ab OR aruba:ti,ab OR azerbaijan:ti,ab OR bahrain:ti,ab OR bangladesh:ti,ab OR barbados:ti,ab OR benin:ti,ab OR byelarus:ti,ab OR byelorussian:ti,ab OR belarus:ti,ab OR belorussian:ti,ab OR belorussia:ti,ab OR belize:ti,ab OR bhutan:ti,ab OR bolivia:ti,ab OR bosnia:ti,ab OR herzegovina:ti,ab OR hercegovina:ti,ab OR botswana:ti,ab OR brasil:ti,ab OR brazil:ti,ab OR bulgaria:ti,ab OR ‘burkina faso’:ti,ab OR ‘burkina fasso’:ti,ab OR ‘upper volta’:ti,ab OR burundi:ti,ab OR urundi:ti,ab OR cambodia:ti,ab OR ‘khmer republic’:ti,ab OR kampuchea:ti,ab OR cameroon:ti,ab OR cameroons:ti,ab OR cameron:ti,ab OR camerons:ti,ab OR ‘cape verde’:ti,ab OR ‘cabo verde’:ti,ab OR ‘central african republic’:ti,ab OR chad:ti,ab OR chile:ti,ab OR china:ti,ab OR colombia:ti,ab OR comoros:ti,ab OR ‘comoro islands’:ti,ab OR comores:ti,ab OR mayotte:ti,ab OR congo:ti,ab OR zaire:ti,ab OR ‘costa rica’:ti,ab OR ‘cote d`ivoire’ OR ‘ivory coast’:ti,ab OR croatia:ti,ab OR cuba:ti,ab OR cyprus:ti,ab OR czechoslovakia:ti,ab OR ‘czech republic’:ti,ab OR slovakia:ti,ab OR ‘slovak republic’:ti,ab OR djibouti:ti,ab OR ‘french somaliland’:ti,ab OR dominica:ti,ab OR ‘dominican republic’:ti,ab OR ‘east timor’:ti,ab OR ‘east timur’:ti,ab OR ‘timor leste’:ti,ab OR ecuador:ti,ab OR egypt:ti,ab OR ‘united arab republic’:ti,ab OR ‘el salvador’:ti,ab OR eritrea:ti,ab OR estonia:ti,ab OR eswatini:ti,ab OR ethiopia:ti,ab OR fiji:ti,ab OR gabon:ti,ab OR ‘gabonese republic’:ti,ab OR gambia:ti,ab OR gaza:ti,ab OR ‘georgia republic’:ti,ab OR ‘georgian republic’:ti,ab OR ghana:ti,ab OR ‘gold coast’:ti,ab OR grenada:ti,ab OR guatemala:ti,ab OR guinea:ti,ab OR guam:ti,ab OR guiana:ti,ab OR guyana:ti,ab OR haiti:ti,ab OR honduras:ti,ab OR india:ti,ab OR maldives:ti,ab OR indonesia:ti,ab OR iran:ti,ab OR iraq:ti,ab OR ‘isle of man’:ti,ab OR jamaica:ti,ab OR jordan:ti,ab OR kazakhstan:ti,ab OR kazakh:ti,ab OR kenya:ti,ab OR kiribati:ti,ab OR korea:ti,ab OR kosovo:ti,ab OR kyrgyzstan:ti,ab OR kirghizia:ti,ab OR ‘kyrgyz republic’:ti,ab OR kirghiz:ti,ab OR kirgizstan:ti,ab OR ‘lao pdr’:ti,ab OR laos:ti,ab OR latvia:ti,ab OR lebanon:ti,ab OR lesotho:ti,ab OR basutoland:ti,ab OR liberia:ti,ab OR libya:ti,ab OR lithuania:ti,ab OR macedonia:ti,ab OR madagascar:ti,ab OR ‘malagasy republic’:ti,ab OR malaysia:ti,ab OR malaya:ti,ab OR malay:ti,ab OR sabah:ti,ab OR sarawak:ti,ab OR malawi:ti,ab OR nyasaland:ti,ab OR mali:ti,ab OR malta:ti,ab OR ‘marshall islands’:ti,ab OR mauritania:ti,ab OR mauritius:ti,ab OR melanesia:ab,ti OR ‘agalega islands’:ti,ab OR mexico:ti,ab OR micronesia:ti,ab OR ‘middle east’:ti,ab OR moldova:ti,ab OR moldovia:ti,ab OR moldovian:ti,ab OR mongolia:ti,ab OR morocco:ti,ab OR ifni:ti,ab OR mozambique:ti,ab OR myanmar:ti,ab OR myanma:ti,ab OR burma:ti,ab OR namibia:ti,ab OR nepal:ti,ab OR ‘netherlands antilles’:ti,ab OR ‘new caledonia’:ti,ab OR nicaragua:ti,ab OR niger:ti,ab OR nigeria:ti,ab OR ‘northern mariana islands’:ti,ab OR oman:ti,ab OR muscat:ti,ab OR pakistan:ti,ab OR palau:ti,ab OR palestine:ti,ab OR panama:ti,ab OR paraguay:ti,ab OR peru:ti,ab OR philippines:ti,ab OR philipines:ti,ab OR phillipines:ti,ab OR phillippines:ti,ab OR poland:ti,ab OR portugal:ti,ab OR ‘puerto rico’:ti,ab OR romania:ti,ab OR rumania:ti,ab OR roumania:ti,ab OR russia:ti,ab OR russian:ti,ab OR rwanda:ti,ab OR ruanda:ti,ab OR ‘saint kitts’:ti,ab OR ‘st kitts’:ti,ab OR nevis:ti,ab OR ‘saint lucia’:ti,ab OR ‘st lucia’:ti,ab OR ‘saint vincent’:ti,ab OR ‘st vincent’:ti,ab OR grenadines:ti,ab OR samoa:ti,ab OR ‘samoan islands’:ti,ab OR ‘navigator island’:ti,ab OR ‘navigator islands’:ti,ab OR ‘sao tome’:ti,ab OR ‘saudi arabia’:ti,ab OR senegal:ti,ab OR serbia:ti,ab OR montenegro:ti,ab OR seychelles:ti,ab OR ‘sierra leone’:ti,ab OR slovenia:ti,ab OR ‘sri lanka’:ti,ab OR ceylon:ti,ab OR ‘solomon islands’:ti,ab OR somalia:ti,ab OR sudan:ti,ab OR suriname:ti,ab OR surinam:ti,ab OR swaziland:ti,ab OR syria:ti,ab OR syrian:ti,ab OR tajikistan:ti,ab OR tadzhikistan:ti,ab OR tadjikistan:ti,ab OR tadzhik:ti,ab OR tanzania:ti,ab OR thailand:ti,ab OR togo:ti,ab OR ‘togolese republic’:ti,ab OR tonga:ti,ab OR trinidad:ti,ab OR tobago:ti,ab OR tunisia:ti,ab OR turkey:ti,ab OR turkmenistan:ti,ab OR turkmen:ti,ab OR tuvalu:ti,ab OR uganda:ti,ab OR ukraine:ti,ab OR uruguay:ti,ab OR ussr:ti,ab OR ‘soviet union’:ti,ab OR ‘union of soviet socialist republics’:ti,ab OR uzbekistan:ti,ab OR uzbek OR vanuatu:ti,ab OR ‘new hebrides’:ti,ab OR venezuela:ti,ab OR vietnam:ti,ab OR ‘viet nam’:ti,ab OR ‘west bank’:ti,ab OR yemen:ti,ab OR yugoslavia:ti,ab OR zambia:ti,ab OR zimbabwe:ti,ab OR rhodesia:ti,ab OR ‘developing country’/exp OR ‘africa’/de OR ‘africa south of the sahara’/de OR ‘north africa’/de OR ‘central africa’/de OR ‘asia’/de OR ‘south asia’/de OR ‘southeast asia’/de OR ‘south america’/de OR ‘central america’/de OR ‘south and central america’/de OR ‘atlantic islands’/de OR ‘caribbean islands’/de OR ‘pacific islands’/de OR ‘indian ocean’/de OR ‘eastern europe’/de OR ‘afghanistan’/exp OR ‘albania’/exp OR ‘algeria’/exp OR ‘american samoa’/exp OR ‘angola’/exp OR ‘antigua and barbuda’/exp OR ‘argentina’/exp OR ‘armenia’/exp OR ‘azerbaijan’/exp OR ‘bahrain’/exp OR ‘bangladesh’/exp OR ‘barbados’/exp OR ‘benin’/exp OR ‘belarus’/exp OR ‘baltic states’/exp OR ‘belize’/exp OR ‘bhutan’/exp OR ‘bolivia’/exp OR ‘bosnia and herzegovina’/exp OR ‘botswana’/exp OR ‘brazil’/exp OR ‘bulgaria’/exp OR ‘burkina faso’/exp OR ‘burundi’/exp OR ‘cambodia’/exp OR ‘cameroon’/exp OR ‘cape verde’/exp OR ‘central african republic’/exp OR ‘chad’/exp OR ‘chile’/exp OR ‘china’/exp OR ‘colombia’/exp OR ‘comoros’/exp OR ‘congo’/exp OR ‘costa rica’/exp OR ‘cote d`ivoire’/exp OR ‘croatia’/exp OR ‘cuba’/exp OR ‘cyprus’/exp OR ‘czechoslovakia’/exp OR ‘czech republic’/exp OR ‘slovakia’/exp OR ‘djibouti’/exp OR ‘democratic republic congo’/exp OR ‘dominica’/exp OR ‘dominican republic’/exp OR ‘timor-leste’/exp OR ‘ecuador’/exp OR ‘egypt’/exp OR ‘el salvador’/exp OR ‘eritrea’/exp OR ‘estonia’/exp OR ‘eswatini’/exp OR ‘ethiopia’/exp OR ‘french guiana’/exp OR ‘fiji’/exp OR ‘gabon’/exp OR ‘gambia’/exp OR ‘georgia (republic)’/exp OR ‘ghana’/exp OR ‘grenada’/exp OR ‘guatemala’/exp OR ‘guinea’/exp OR ‘guinea bissau’/exp OR ‘guam’/exp OR ‘guyana’/exp OR ‘haiti’/exp OR ‘honduras’/exp OR ‘india’/exp OR ‘indonesia’/exp OR ‘iran’/exp OR ‘iraq’/exp OR ‘jamaica’/exp OR ‘jordan’/exp OR ‘kazakhstan’/exp OR ‘kenya’/exp OR ‘korea’/exp OR ‘kyrgyzstan’/exp OR ‘laos’/exp OR ‘latvia’/exp OR ‘lebanon’/exp OR ‘lesotho’/exp OR ‘liberia’/exp OR ‘libyan arab jamahiriya’/exp OR ‘lithuania’/exp OR ‘macedonia (republic)’/exp OR ‘madagascar’/exp OR ‘malaysia’/exp OR ‘malawi’/exp OR ‘mali’/exp OR ‘malta’/exp OR ‘mauritania’/exp OR ‘mauritius’/exp OR ‘melanesia’/exp OR ‘mexico’/exp OR ‘federated states of micronesia’/exp OR ‘middle east’/de OR ‘moldova’/exp OR ‘mongolia’/exp OR ‘montenegro’/exp OR ‘morocco’/exp OR ‘mozambique’/exp OR ‘myanmar’/exp OR ‘namibia’/exp OR ‘nepal’/exp OR ‘netherlands antilles’/exp OR ‘new caledonia’/exp OR ‘nicaragua’/exp OR ‘niger’/exp OR ‘nigeria’/exp OR ‘north korea’/exp OR ‘oman’/exp OR ‘pakistan’/exp OR ‘palau’/exp OR ‘panama’/exp OR ‘papua new guinea’/exp OR ‘paraguay’/exp OR ‘peru’/exp OR ‘philippines’/exp OR ‘poland’/exp OR ‘portugal’/exp OR ‘puerto rico’/exp OR ‘romania’/exp OR ‘russian federation’/exp OR ‘rwanda’/exp OR ‘saint kitts and nevis’/exp OR ‘saint lucia’/exp OR ‘saint vincent and the grenadines’/exp OR ‘samoan islands’/exp OR ‘samoa’/exp OR ‘saudi arabia’/exp OR ‘senegal’/exp OR ‘serbia’/exp OR ‘montenegro (republic)’/exp OR ‘seychelles’/exp OR ‘sierra leone’/exp OR ‘slovenia’/exp OR ‘sri lanka’/exp OR ‘somalia’/exp OR ‘south korea’/exp OR ‘south africa’/exp OR ‘sudan’/exp OR ‘suriname’/exp OR ‘swaziland’/exp OR ‘syrian arab republic’/exp OR ‘tajikistan’/exp OR ‘tanzania’/exp OR ‘thailand’/exp OR ‘togo’/exp OR ‘tonga’/exp OR ‘trinidad and tobago’/exp OR ‘tunisia’/exp OR ‘turkey (republic)’/exp OR ‘turkmenistan’/exp OR ‘uganda’/exp OR ‘ukraine’/exp OR ‘uruguay’/exp OR ‘ussr’/exp OR ‘uzbekistan’/exp OR ‘vanuatu’/exp OR ‘venezuela’/exp OR ‘viet nam’/exp OR ‘yemen’/exp OR ‘yugoslavia’/exp OR ‘yugoslavia (pre-1992)’/exp OR ‘zambia’/exp OR ‘zimbabwe’/exp
**Global Index Medicus Search terms:**
***HPV vaccination/immunization***
D20.215.894.899.498—**search in subject descriptor**
OR
(hpv AND vaccination) OR (hpv AND immunization) OR (hpv AND immunization) OR (hpv AND vaccinations) OR (hpv AND immunisations) OR (hpv AND immunizations) OR ((HPV OR “papillomavirus vaccine” OR “papillomavirus vaccines” OR “papilloma virus vaccine” OR “papilloma virus vaccines” OR Gardasil OR Gardasil9 OR Cervarix OR Cecolin OR Walrinvax OR Cervava) AND (“vaccine delivery” OR “administration” OR “strategy” OR strategies OR implementation)) OR ((HPV OR “human papillomavirus” OR “human papillomaviruses” OR “human papilloma virus” OR “human papilloma viruses”) AND (“immunisation program” OR “immunization program” OR “immunisation programme” OR “immunization programme” OR “immunisation programs” OR “immunization programs” OR “immunisation programmes” OR “immunization programmes”))—***Search in “title, abstract, subject”***

***OOS girls***
“non-school based” OR “nonschool based” OR unschooled OR (“out of” AND school) OR (“out of” AND schools) OR “outreach” OR “out reach” OR (facility AND based) OR “unenrolled” OR “un-enrolled” OR “hard to reach***”***—***Search in “title, abstract, subject”***

***LMICs***
Because this source is all produced in LMICs this is not needed. Also, there were only 13 total results, with 5 in English.

**Scopus Search terms:**
***HPV vaccination/immunization***
TITLE-ABS-KEY (((hpv OR “papillomavirus vaccine*” OR “papilloma virus vaccine*” OR gardasil* OR cervarix OR cecolin OR walrinvax OR cervava*) AND (“vaccine delivery” OR “administration” OR “strateg*” OR implementation)) OR ((hpv OR “human papillomavirus*” OR “human papilloma virus*”) AND (“immunisation program*” OR “immunization program*”)))))

***
  OOS girls
  ***
((TITLE-ABS-KEY (“not” W/3 “school based”) OR TITLE-ABS-KEY (“out of” W/3 school*) OR TITLE-ABS-KEY (facility W/3 based) OR TITLE-ABS-KEY (“non-school based” OR “nonschool based” OR unschooled OR “outreach” OR “out reach” OR “unenrolled” OR “un-enrolled” OR “hard to reach”)))

***LMICs***
TITLE-ABS-KEY(“Antigua and Barbuda “ OR “ Atlantic Islands “ OR “ Baltic States “ OR “ Commonwealth of Independent States “ OR “ Democratic People’s Republic of Korea “ OR “ Democratic Republic of the Congo “ OR “ deprived countries “ OR “ deprived population “ OR “ deprived populations “ OR “ developing countries “ OR “ developing country “ OR “ developing economies “ OR “ developing economy “ OR “ developing nation “ OR “ developing nations “ OR “ developing population “ OR “ developing populations “ OR “ developing world “ OR “ Equatorial Guinea “ OR “ French Guiana “ OR “ Georgia Republic “ OR “ Independent State of Samoa “ OR “ Indian Ocean Islands “ OR “ lami countries “ OR “ lami country “ OR “ less developed countries “ OR “ less developed country “ OR “ less developed economies “ OR “ less developed economy “ OR “ less developed nation “ OR “ less developed nations “ OR “ less developed world “ OR “ lesser developed countries “ OR “ lesser developed nations “ OR “ low gdp “ OR “ low gnp “ OR “ low gross domestic “ OR “ low gross national “ OR “ low income countries “ OR “ low income country “ OR “ low income economies “ OR “ low income economy “ OR “ low income nations “ OR “ low income population “ OR “ low income populations “ OR “ lower gdp “ OR “ lower gross domestic “ OR “ lower income countries “ OR “ lower income country “ OR “ lower income nations “ OR “ lower income population “ OR “ lower income populations “ OR “ Macedonia Republic “ OR “ Melanesia “ OR “ middle income countries “ OR “ middle income country “ OR “ middle income economies “ OR “ middle income nation “ OR “ middle income nations “ OR “ middle income population “ OR “ middle income populations “ OR “ Pacific Islands “ OR “ poor countries “ OR “ poor country “ OR “ poor nation “ OR “ poor nations “ OR “ poor population “ OR “ poor populations “ OR “ poor world “ OR “ poorer countries “ OR “ poorer nations “ OR “ poorer population “ OR “ poorer populations “ OR “ Republic of Belarus “ OR “ Saint Kitts and Nevis “ OR “ Saint Vincent and the Grenadines “ OR “ South Sudan “ OR “ third world “ OR “ transitional countries “ OR “ transitional country “ OR “ Trinidad and Tobago “ OR “ under developed countries “ OR “ under developed country “ OR “ under developed nations “ OR “ under developed world “ OR “ under served population “ OR “ under served populations “ OR “ underdeveloped countries “ OR “ underdeveloped country “ OR “ underdeveloped economies “ OR “ underdeveloped nations “ OR “ underdeveloped population “ OR “ underdeveloped world “ OR “ underserved countries “ OR “ underserved nations “ OR “ underserved population “ OR “ underserved populations “ OR “ Afghanistan “ OR “ Africa “ OR “ Albania “ OR “ Algeria “ OR “ American Samoa “ OR “ Angola “ OR “ Argentina “ OR “ Armenia “ OR “ Asia “ OR “ Azerbaijan “ OR “ Bahrain “ OR “ Bangladesh “ OR “ Barbados “ OR “ Belize “ OR “ Benin “ OR “ Bhutan “ OR “ Bolivia “ OR “ Bosnia-Herzegovina “ OR “ Botswana “ OR “ Brazil “ OR “ Bulgaria “ OR “ Burkina Faso “ OR “ Burundi “ OR “ Cambodia “ OR “ Cameroon “ OR “ Cape Verde “ OR “ Caribbean Region “ OR “ Central African Republic “ OR “ Central America “ OR “ Chad “ OR “ Chile “ OR “ China “ OR “ Colombia “ OR “ Comoros “ OR “ Congo “ OR “ Costa Rica “ OR “ Cote d’Ivoire “ OR “ Croatia “ OR “ Cuba “ OR “ Cyprus “ OR “ Czech Republic “ OR “ Czechoslovakia “ OR “ Developing Countries “ OR “ Djibouti “ OR “ Dominica “ OR “ Dominican Republic “ OR “ East Timor “ OR “ Ecuador “ OR “ Egypt “ OR “ El Salvador “ OR “ Eritrea “ OR “ Estonia “ OR “ Ethiopia “ OR “ Fiji “ OR “ Gabon “ OR “ Gambia “ OR “ Ghana “ OR “ Greece “ OR “ Grenada “ OR “ Guam “ OR “ Guatemala “ OR “ Guinea “ OR “ Guinea-Bissau “ OR “ Guyana “ OR “ Haiti “ OR “ Honduras “ OR “ Hungary “ OR “ India “ OR “ Indonesia “ OR “ Iran “ OR “ Iraq “ OR “ Jamaica “ OR “ Jordan “ OR “ Kazakhstan “ OR “ Kenya “ OR “ Korea “ OR “ Kyrgyzstan “ OR “ Laos “ OR “ Latin America “ OR “ Latvia “ OR “ Lebanon “ OR “ Lesotho “ OR “ Liberia “ OR “ Libya “ OR “ Lithuania “ OR “ lmic “ OR “ lmics “ OR “ Madagascar “ OR “ Malawi “ OR “ Malaysia “ OR “ Mali “ OR “ Malta “ OR “ Mauritania “ OR “ Mauritius “ OR “ Mexico “ OR “ Micronesia “ OR “ Middle East “ OR “ Moldova “ OR “ Mongolia “ OR “ Montenegro “ OR “ Montenegro “ OR “ Morocco “ OR “ Mozambique “ OR “ Myanmar “ OR “ Namibia “ OR “ Nepal “ OR “ Netherlands Antilles “ OR “ New Caledonia “ OR “ Nicaragua “ OR “ Niger “ OR “ Nigeria “ OR “ Oman “ OR “ Pakistan “ OR “ Palau “ OR “ Panama “ OR “ Papua New Guinea “ OR “ Paraguay “ OR “ Peru “ OR “ Philippines “ OR “ Poland “ OR “ Portugal “ OR “ Puerto Rico “ OR “ Romania “ OR “ Russia “ OR “ Rwanda “ OR “ Saint Lucia “ OR “ Samoa “ OR “ Saudi Arabia “ OR “ Senegal “ OR “ Serbia “ OR “ Seychelles “ OR “ Sierra Leone “ OR “ Slovakia “ OR “ Slovenia “ OR “ Somalia “ OR “ South Africa “ OR “ South America “ OR “ Sri Lanka “ OR “ Sudan “ OR “ Suriname “ OR “ Swaziland “ OR “ Syria “ OR “ Tajikistan “ OR “ Tanzania “ OR “ Thailand “ OR “ Togo “ OR “ Tonga “ OR “ Tunisia “ OR “ Turkey “ OR “ Turkmenistan “ OR “ Uganda “ OR “ Ukraine “ OR “ Uruguay “ OR “ USSR “ OR “ Uzbekistan “ OR “ Vanuatu “ OR “ Venezuela “ OR “ Vietnam “ OR “ West Indies “ OR “ Yemen “ OR “ Yugoslavia “ OR “ Zambia “ OR “ Zimbabwe”)
OR
***Cut and paste into Advanced Search:***
(TITLE-ABS-KEY (“Antigua and Barbuda “ OR “ Atlantic Islands “ OR “ Baltic States “ OR “ Commonwealth of Independent States “ OR “ Democratic People’s Republic of Korea “ OR “ Democratic Republic of the Congo “ OR “ deprived countries “ OR “ deprived population “ OR “ deprived populations “ OR “ developing countries “ OR “ developing country “ OR “ developing economies “ OR “ developing economy “ OR “ developing nation “ OR “ developing nations “ OR “ developing population “ OR “ developing populations “ OR “ developing world “ OR “ Equatorial Guinea “ OR “ French Guiana “ OR “ Georgia Republic “ OR “ Independent State of Samoa “ OR “ Indian Ocean Islands “ OR “ lami countries “ OR “ lami country “ OR “ less developed countries “ OR “ less developed country “ OR “ less developed economies “ OR “ less developed economy “ OR “ less developed nation “ OR “ less developed nations “ OR “ less developed world “ OR “ lesser developed countries “ OR “ lesser developed nations “ OR “ low gdp “ OR “ low gnp “ OR “ low gross domestic “ OR “ low gross national “ OR “ low income countries “ OR “ low income country “ OR “ low income economies “ OR “ low income economy “ OR “ low income nations “ OR “ low income population “ OR “ low income populations “ OR “ lower gdp “ OR “ lower gross domestic “ OR “ lower income countries “ OR “ lower income country “ OR “ lower income nations “ OR “ lower income population “ OR “ lower income populations “ OR “ Macedonia Republic “ OR “ Melanesia “ OR “ middle income countries “ OR “ middle income country “ OR “ middle income economies “ OR “ middle income nation “ OR “ middle income nations “ OR “ middle income population “ OR “ middle income populations “ OR “ Pacific Islands “ OR “ poor countries “ OR “ poor country “ OR “ poor nation “ OR “ poor nations “ OR “ poor population “ OR “ poor populations “ OR “ poor world “ OR “ poorer countries “ OR “ poorer nations “ OR “ poorer population “ OR “ poorer populations “ OR “ Republic of Belarus “ OR “ Saint Kitts and Nevis “ OR “ Saint Vincent and the Grenadines “ OR “ South Sudan “ OR “ third world “ OR “ transitional countries “ OR “ transitional country “ OR “ Trinidad and Tobago “ OR “ under developed countries “ OR “ under developed country “ OR “ under developed nations “ OR “ under developed world “ OR “ under served population “ OR “ under served populations “ OR “ underdeveloped countries “ OR “ underdeveloped country “ OR “ underdeveloped economies “ OR “ underdeveloped nations “ OR “ underdeveloped population “ OR “ underdeveloped world “ OR “ underserved countries “ OR “ underserved nations “ OR “ underserved population “ OR “ underserved populations “ OR “ Afghanistan “ OR “ Africa “ OR “ Albania “ OR “ Algeria “ OR “ American Samoa “ OR “ Angola “ OR “ Argentina “ OR “ Armenia “ OR “ Asia “ OR “ Azerbaijan “ OR “ Bahrain “ OR “ Bangladesh “ OR “ Barbados “ OR “ Belize “ OR “ Benin “ OR “ Bhutan “ OR “ Bolivia “ OR “ Bosnia-Herzegovina “ OR “ Botswana “ OR “ Brazil “ OR “ Bulgaria “ OR “ Burkina Faso “ OR “ Burundi “ OR “ Cambodia “ OR “ Cameroon “ OR “ Cape Verde “ OR “ Caribbean Region “ OR “ Central African Republic “ OR “ Central America “ OR “ Chad “ OR “ Chile “ OR “ China “ OR “ Colombia “ OR “ Comoros “ OR “ Congo “ OR “ Costa Rica “ OR “ Cote d’Ivoire “ OR “ Croatia “ OR “ Cuba “ OR “ Cyprus “ OR “ Czech Republic “ OR “ Czechoslovakia “ OR “ Developing Countries “ OR “ Djibouti “ OR “ Dominica “ OR “ Dominican Republic “ OR “ East Timor “ OR “ Ecuador “ OR “ Egypt “ OR “ El Salvador “ OR “ Eritrea “ OR “ Estonia “ OR “ Ethiopia “ OR “ Fiji “ OR “ Gabon “ OR “ Gambia “ OR “ Ghana “ OR “ Greece “ OR “ Grenada “ OR “ Guam “ OR “ Guatemala “ OR “ Guinea “ OR “ Guinea-Bissau “ OR “ Guyana “ OR “ Haiti “ OR “ Honduras “ OR “ Hungary “ OR “ India “ OR “ Indonesia “ OR “ Iran “ OR “ Iraq “ OR “ Jamaica “ OR “ Jordan “ OR “ Kazakhstan “ OR “ Kenya “ OR “ Korea “ OR “ Kyrgyzstan “ OR “ Laos “ OR “ Latin America “ OR “ Latvia “ OR “ Lebanon “ OR “ Lesotho “ OR “ Liberia “ OR “ Libya “ OR “ Lithuania “ OR “ lmic “ OR “ lmics “ OR “ Madagascar “ OR “ Malawi “ OR “ Malaysia “ OR “ Mali “ OR “ Malta “ OR “ Mauritania “ OR “ Mauritius “ OR “ Mexico “ OR “ Micronesia “ OR “ Middle East “ OR “ Moldova “ OR “ Mongolia “ OR “ Montenegro “ OR “ Montenegro “ OR “ Morocco “ OR “ Mozambique “ OR “ Myanmar “ OR “ Namibia “ OR “ Nepal “ OR “ Netherlands Antilles “ OR “ New Caledonia “ OR “ Nicaragua “ OR “ Niger “ OR “ Nigeria “ OR “ Oman “ OR “ Pakistan “ OR “ Palau “ OR “ Panama “ OR “ Papua New Guinea “ OR “ Paraguay “ OR “ Peru “ OR “ Philippines “ OR “ Poland “ OR “ Portugal “ OR “ Puerto Rico “ OR “ Romania “ OR “ Russia “ OR “ Rwanda “ OR “ Saint Lucia “ OR “ Samoa “ OR “ Saudi Arabia “ OR “ Senegal “ OR “ Serbia “ OR “ Seychelles “ OR “ Sierra Leone “ OR “ Slovakia “ OR “ Slovenia “ OR “ Somalia “ OR “ South Africa “ OR “ South America “ OR “ Sri Lanka “ OR “ Sudan “ OR “ Suriname “ OR “ Swaziland “ OR “ Syria “ OR “ Tajikistan “ OR “ Tanzania “ OR “ Thailand “ OR “ Togo “ OR “ Tonga “ OR “ Tunisia “ OR “ Turkey “ OR “ Turkmenistan “ OR “ Uganda “ OR “ Ukraine “ OR “ Uruguay “ OR “ USSR “ OR “ Uzbekistan “ OR “ Vanuatu “ OR “ Venezuela “ OR “ Vietnam “ OR “ West Indies “ OR “ Yemen “ OR “ Yugoslavia “ OR “ Zambia “ OR “ Zimbabwe”)) AND ((TITLE-ABS-KEY ((hpv W/3 vaccinat*) OR (hpv W/3 immuni*)) OR TITLE-ABS-KEY (((hpv OR “papillomavirus vaccine*” OR “papilloma virus vaccine*” OR gardasil* OR cervarix OR cecolin OR walrinvax OR cervava*) AND (“vaccine delivery” OR “administration” OR “strateg*” OR implementation)) OR ((hpv OR “human papillomavirus*” OR “human papilloma virus*”) AND (“immuni?ation program*” OR “immunization program*”))))) AND ((TITLE-ABS-KEY (“not” W/3 “school based”) OR TITLE-ABS-KEY (“out of” W/3 school*) OR TITLE-ABS-KEY (facility W/3 based) OR TITLE-ABS-KEY (“non-school based” OR “nonschool based” OR unschooled OR “outreach” OR “out reach” OR “unenrolled” OR “un-enrolled” OR “hard to reach”)))

*Inclusion and Exclusion Criteria*
**
  Inclusion criteria
  **

- Published in English
- Low-income, low-middle income, or middle-income country/countries setting
- Target population includes OOS girls or hard-to-reach populations
- Article must report on a discrete service delivery strategy to reach OOS girls.

**
  Exclusion criteria
  **

- Non-English language publication
- Publication is **not** featured in an academic peer-reviewed journal
- The article is a description of a review, protocol, commentary/advocacy pieces, meeting abstract, proceeding paper, news media items, book chapters, corrections, book review, biographical item, poem, retraction.
- Study is a randomized trial addressing vaccine-dosing regimens
- Studies presenting non-empirical (i.e., simulated or modeled) data
- Studies that are only descriptive in nature with no intervention implemented or assessed
- No reference to reaching OOS populations/hard-to-reach populations.
